# A Clip-Based Dairy Cow Behavior Recognition Method Integrating Temporal Modeling and Behavioral Priors

**DOI:** 10.3390/ani16132087

**Published:** 2026-07-06

**Authors:** Xiaoying Li, Huijuan Wu, Daoerji Fan, Jiaqi Bai, Chunyun Wang, Yan Liu

**Affiliations:** 1 School of Electronic Information Engineering, Inner Mongolia University, No. 235 College Road, Hohhot 010021, China; 32456031@mail.imu.edu.cn (X.L.); fandaoerji@imu.edu.cn (D.F.); 32456076@mail.imu.edu.cn (J.B.); 32456035@mail.imu.edu.cn (C.W.); 32456061@mail.imu.edu.cn (Y.L.); 2 Inner Mongolia Key Laboratory of Intelligent Communication and Sensing and Signal Processing, Inner Mongolia University, No. 235 College Road, Hohhot 010021, China

**Keywords:** dairy cow behavior recognition, precision livestock farming, CLIP visual encoder, temporal modeling, behavioral priors, non-contact video monitoring

## Abstract

Accurate dairy cow behavior recognition is important for health monitoring, welfare assessment, and early warning in smart livestock farming. However, recognizing fine-grained behaviors such as feeding, drinking, and rumination remains difficult in real barns because of occlusion, complex backgrounds, subtle motion changes, and class imbalance. This study proposes a behavior recognition method that integrates temporal modeling and behavioral priors. The Contrastive Language–Image Pre-training (CLIP) visual encoder is used as the feature extraction backbone, while two temporal adapters are introduced to model dynamic information across consecutive video frames. Dairy cow behavior recognition is further decoupled into posture recognition and action recognition, and a behavioral prior loss is designed to softly constrain unlikely posture–action combinations, such as lying with feeding or lying with drinking. On the test set, the proposed method achieves a five-class accuracy of 75.45%, a five-class Macro-F1 of 0.7246, and an Action Macro-F1 of 0.7605, outperforming the CLIP baseline and several representative video recognition models. These results indicate that the proposed method can support non-contact monitoring of key dairy cow behaviors for practical barn management.

## Simple Summary

Dairy cows show changes in behavior when their health, comfort, or feeding condition changes. Recognizing behaviors such as standing, lying, feeding, drinking, and rumination can therefore help farmers detect possible problems earlier and improve daily herd management. However, in real barns, automatic behavior recognition is difficult because cows may block each other, the background is complex, and some actions look very similar. This study develops a video-based method for recognizing dairy cow behavior without attaching sensors to the animals. The method learns both body posture and action information and uses common biological knowledge about which posture and action combinations are reasonable. For example, feeding and drinking are usually associated with a standing posture, whereas lying with feeding or drinking is unlikely in normal barn conditions. By combining video information over time with this behavioral knowledge, the proposed method improves recognition accuracy and produces results that are easier to interpret. This work supports non-contact monitoring for animal health, welfare assessment, and practical dairy farm management.

## 1. Introduction

Cattle farming constitutes an important component of global agricultural production, and dairy cows are central to the dairy supply system. Improving animal health, welfare, and production efficiency is also relevant to maintaining a stable food supply and supporting more sustainable livestock systems [1]. With the development of precision livestock farming and smart barns, dairy cow behavior has increasingly been regarded as an important indicator of health status, feeding condition, production performance, and animal welfare [2,3,4]. More broadly, intelligent connected systems and edge/cloud task allocation have become important technical foundations for deploying AI-based monitoring models in real environments [5]. Continuous and accurate recognition of key behaviors, including standing, lying, feeding, drinking, and rumination, can provide useful evidence for early disease warning, welfare assessment, and daily herd management [6,7].

However, video-based dairy cow behavior recognition remains challenging under real barn conditions. On the one hand, barn scenes usually involve complex backgrounds, illumination variations, facility occlusion, and mutual occlusion among multiple cows. On the other hand, the visual differences among fine-grained behaviors are often subtle. For example, feeding, drinking, and rumination may all involve head lowering or weak mouth movements, making them difficult to distinguish using single-frame appearance features alone. In addition, standing and lying mainly describe posture states, whereas feeding, drinking, and rumination depend more on action processes. Treating all behaviors simply as mutually exclusive categories may therefore ignore the biological relationship between posture states and action behaviors.

Dairy cow behavior datasets are generally much smaller than general action-recognition datasets, making large video models trained from scratch susceptible to overfitting. The CLIP visual encoder was therefore adopted to provide transferable frame-level representations while keeping most backbone parameters frozen. However, because CLIP is pretrained on static image–text pairs, it lacks explicit temporal modeling, which motivates the introduction of lightweight temporal adapters.

Based on the above challenges, the motivation of this study can be summarized from three aspects. First, stronger temporal modeling is required because fine-grained dairy cow behaviors are often reflected in short-term and local dynamic changes rather than in static appearance alone. Second, behavior recognition methods should be compatible with practical barn deployment; therefore, non-contact video recognition with limited additional training cost is more suitable for long-term herd monitoring than labor-intensive manual observation or intrusive sensing, which is consistent with the welfare-oriented development of precision livestock farming [8,9]. Third, dairy cow behaviors contain inherent posture–action relationships, since standing and lying describe body states, whereas feeding, drinking, and rumination describe action processes. Introducing such behavioral relationships into model design is therefore expected to improve the rationality and interpretability of recognition results. Motivated by these considerations, this study proposes a dairy cow behavior recognition method integrating temporal modeling and behavioral priors.

## 2. Related Work

### 2.1. Dairy Cow and Video Behavior Recognition

Dairy cow behavior recognition is an important task in precision livestock farming and smart barns. Existing studies have used wearable sensors, vision-based methods, and multimodal fusion to recognize feeding, drinking, rumination, lying, and related behaviors [10,11,12,13,14]. Although wearable sensors can provide stable motion signals, device attachment, maintenance cost, and potential animal stress limit their long-term use in large herds [15]. Video-based recognition is therefore attractive for fixed barn cameras because it enables non-contact monitoring under natural conditions. However, barn videos are affected by occlusion, illumination variation, complex backgrounds, and multi-animal interference [16,17,18]. General video recognition models, including three-dimensional convolutional networks, SlowFast networks, masked video pretraining, and video Transformers, have shown the value of temporal modeling [19,20,21,22,23,24], but they are mostly developed for large-scale human action datasets and usually require substantial trainable parameters. In contrast, dairy cow behavior datasets are smaller, and fine-grained behaviors such as feeding, drinking, and rumination often differ only in weak head or mouth movements. These characteristics motivate a lighter temporal modeling strategy tailored to barn-based dairy cow behavior recognition.

Evaluation protocols also influence the interpretation of dairy cow behavior recognition results. For example, the CBVD-5 baseline randomly divided processed samples into training, validation, and test sets at a ratio of 70:20:10 [25], whereas Bai et al. used an 80:20 stratified split for a dairy cow video dataset [26]. Such protocols are useful for benchmarking, but random frame- or clip-level partitioning may place visually similar samples from the same source video in different subsets. Therefore, results obtained under frame-, clip-, video-, or animal-level splits should not be compared without considering data independence. In this study, source-video-level partitioning was performed before clip generation to avoid leakage among clips from the same video, although animal-level independence could not be verified because persistent cow identities were unavailable in the released annotations.

### 2.2. CLIP-Based Video Adaptation and Parameter-Efficient Fine-Tuning

CLIP learns transferable visual representations through large-scale image–text contrastive pretraining and has shown strong generalization in downstream recognition tasks [27]. Existing CLIP-based video methods introduce temporal aggregation or lightweight adapters to extend static visual representations to action recognition [28,29,30], while parameter-efficient approaches reduce adaptation cost by freezing most pretrained parameters [31,32]. Nevertheless, these methods mainly focus on general human actions and do not explicitly consider the subtle local movements or posture–action relationships of dairy cows. In this study, TED-Adapter models token-level temporal changes and adjacent-frame differences, whereas FTE-Adapter and hybrid temporal pooling enhance frame-level dynamics. A soft behavioral prior is further introduced to exploit domain-specific posture–action relationships, distinguishing the proposed method from general CLIP video adaptation approaches.

### 2.3. Prior Knowledge Constraints in Behavior Recognition

Purely data-driven deep models can learn discriminative visual features, but their predictions may still be inconsistent with domain knowledge when the data are imbalanced or visual evidence is ambiguous. In broader machine learning studies, prior knowledge has been incorporated through logical constraints, semantic regularization, and neuro-symbolic learning to improve prediction consistency and interpretability [33,34]. For dairy cow behavior recognition, such prior knowledge is biologically meaningful because posture states and action categories are not arbitrary combinations. Feeding and drinking usually occur in a standing posture, rumination can occur while standing or lying, and lying with feeding or lying with drinking is unlikely under normal barn conditions. Existing single-output classification frameworks tend to treat standing, lying, feeding, drinking, and rumination as mutually exclusive labels, which ignores this compositional relationship. Therefore, a posture–action dual-head formulation and a differentiable behavioral prior loss are introduced in this study so that unlikely posture–action combinations can be softly constrained during training rather than corrected only by hard post-processing after inference.

## 3. Materials and Methods

### 3.1. Baseline Model

To provide a clear reference for evaluating the proposed temporal modeling and behavioral prior modules, a baseline model was first constructed using the CLIP visual encoder, temporal average pooling, and a dual-branch classifier. In this baseline, only the visual encoder of CLIP ViT-B/16 was used as the frame-level feature extraction backbone, whereas the text encoder and the image–text similarity matching mechanism were not used. For each input video clip, a fixed number of frames was uniformly sampled and each frame was independently fed into the CLIP visual encoder. The class token of each frame was extracted as the frame-level global visual feature.

**Figure 1:**
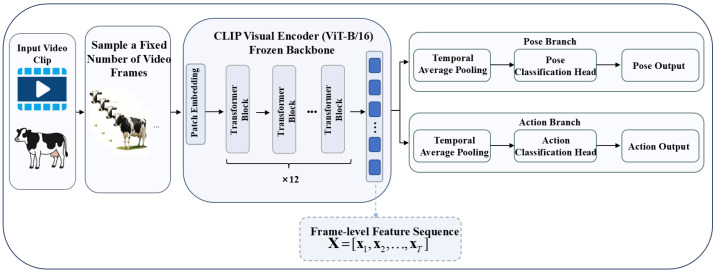
Architecture of the baseline model.

The baseline architecture is shown in Figure 1. It provides a reference model in which frame-level CLIP visual features are aggregated by temporal average pooling and then fed into separate posture and action classification heads. No temporal adapter, hybrid temporal pooling module, or behavioral prior constraint is included in this baseline.

Let the sampled video clip be denoted as $V=\{I_{1},I_{2},\ldots ,I_{T}\}$, where *T* denotes the number of sampled frames. The frame-level feature extracted by the CLIP visual encoder can be written as

(1) $$x_{t}=E_{\mathrm{CLIP}}\left(I_{t}\right),\;t=1,2,\ldots ,T,$$

where $E_{\mathrm{CLIP}}\left(\cdot \right)$ denotes the CLIP visual encoder and $x_{t}$ denotes the class-token feature of the *t*-th frame. Temporal average pooling was then applied to aggregate the frame-level features into a video-level representation:

(2) $$x_{\mathrm{base}}=\frac{1}{T}\sum_{t=1}^{T}x_{t}.$$

Unlike a conventional single-output five-class classifier, the baseline adopted a dual-branch classification structure. The posture branch predicted standing or lying, whereas the action branch predicted none, feeding, drinking, or rumination:

(3) $$p_{\mathrm{base}}^{p}=\mathrm{Softmax}\left(W_{p}^{b}x_{\mathrm{base}}+b_{p}^{b}\right),\;p_{\mathrm{base}}^{a}=\mathrm{Softmax}\left(W_{a}^{b}x_{\mathrm{base}}+b_{a}^{b}\right).$$

This baseline retains the posture–action decoupled classification form, but does not include TED-Adapter, FTE-Adapter, hybrid temporal pooling, or the behavioral prior loss. Therefore, it was used to evaluate the performance obtained from CLIP frame-level visual representation, simple temporal average pooling, and the dual-branch classifier itself. In this way, the baseline was designed to isolate the contribution of the proposed temporal modeling and behavioral prior modules while maintaining the same posture–action decoupled classification framework.

### 3.2. Proposed Model

**Figure 2:**
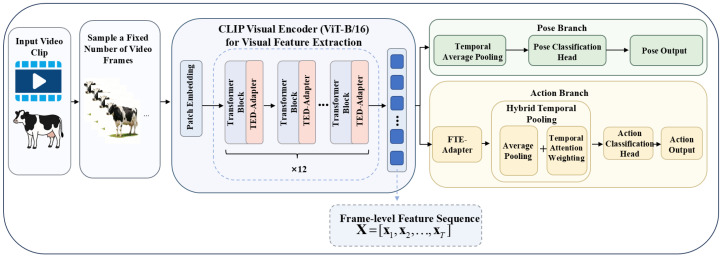
Overall framework of the proposed dairy cow behavior recognition method.

Based on the dual-head CLIP baseline, a dairy cow behavior recognition framework integrating temporal modeling and behavioral priors was proposed. As shown in Figure 2, the proposed model retained the CLIP visual encoder and the posture–action dual-head formulation used in the baseline, while TED-Adapter, FTE-Adapter, hybrid temporal pooling, and a posture–action behavioral prior loss were further introduced. These additional modules were designed to strengthen temporal representation across consecutive video frames and reduce selected low-probability posture–action combinations through soft behavioral constraints. In this way, posture states and action behaviors were still modeled as two correlated subtasks, whereas the improvement over the baseline mainly came from enhanced temporal modeling and behavioral prior constraints.

Let the input video clip be denoted as *V*, with tensor form $V\in R^{B\times C\times T\times H\times W}$, where *B* denotes the batch size, *C* denotes the number of image channels, *T* denotes the number of sampled frames, and *H* and *W* denote the height and width of the input image, respectively. For each frame, the image was first divided by the CLIP visual encoder into fixed-size patches, which were then projected into a sequence of visual tokens. A class token was prepended to the patch token sequence, and positional embeddings were added to produce the global visual representation of each frame.

For task formulation, standing, lying, feeding, drinking, and rumination were not directly treated as a single set of mutually exclusive classes. Instead, behavior recognition was decomposed into posture recognition and action recognition. The posture branch was used to predict whether the cow was standing or lying, whereas the action branch was used to predict whether no obvious action, feeding, drinking, or rumination was present. The model outputs are defined as

(4) $$p^{p}=\mathrm{Softmax}\left(z^{p}\right),\;p^{a}=\mathrm{Softmax}\left(z^{a}\right),$$

where $p^{p}$ and $p^{a}$ denote the posture and action probability distributions, respectively.

#### 3.2.1. Temporal Modeling Modules

Because the CLIP visual encoder was originally designed for single-frame image representation, additional temporal modeling is required for video-based behavior recognition. Two lightweight modules were therefore introduced. TED-Adapter was inserted into the CLIP visual Transformer blocks to model token-level temporal enhancement and adjacent-frame differences, whereas FTE-Adapter was used in the action branch to enhance short-term dependencies among frame-level class-token features.

**Figure 3:**
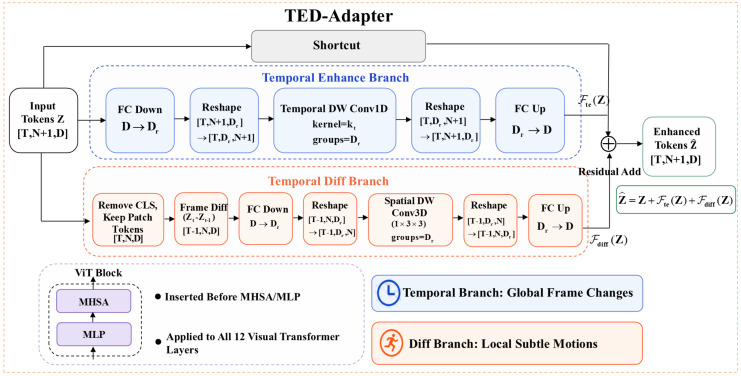
Structure of the TED-Adapter.

As illustrated in Figure 3, TED-Adapter was inserted before both the multi-head self-attention module and the feed-forward network module in all 12 visual Transformer blocks of CLIP ViT-B/16. The token dimension was reduced from 768 to a 384-dimensional bottleneck and then restored to 768 after temporal processing. Given the input token feature Z, TED-Adapter is expressed in residual form as

(5) $$\hat{Z}=Z+F_{\mathrm{te}}\left(Z\right)+F_{\mathrm{diff}}\left(Z\right),$$

where $F_{\mathrm{te}}\left(\cdot \right)$ denotes the temporal enhancement branch and $F_{\mathrm{diff}}\left(\cdot \right)$ denotes the adjacent-frame difference branch. In the temporal enhancement branch, the token tensor was reshaped from [B×T,L,C] to $\left[B\times L,C_{r},T\right]$, where $C_{r}=384$. A depthwise one-dimensional convolution with kernel size 3, stride 1, padding 1, and 384 groups was applied along the temporal dimension, after which the features were reshaped back and projected to 768 dimensions:

(6) $$F_{\mathrm{te}}\left(Z\right)=W_{\mathrm{up}}\mathrm{Conv}1D\left(W_{\mathrm{down}}Z\right).$$

Here, Conv1D models short-term frame-to-frame dynamics, and $W_{\mathrm{down}}$ and $W_{\mathrm{up}}$ denote the learnable down-projection and up-projection parameters.

In the adjacent-frame difference branch, the class token was excluded and differences were calculated between corresponding patch tokens in adjacent frames. The difference tensor was projected into the same 384-dimensional bottleneck space and reshaped to $\left[B,C_{r},T,H_{p},W_{p}\right]$, where $H_{p}$ and $W_{p}$ denote the spatial height and width of the patch-token grid. A depthwise three-dimensional convolution with a kernel of 1×3×3, stride 1, padding (0,1,1), and 384 groups was then used:

(7) $$F_{\mathrm{diff}}\left(Z_{t}\right)=\mathrm{Conv}3D\left(Z_{t}-Z_{t-1}\right).$$

This branch emphasizes local motion cues after adjacent-frame differencing, especially subtle changes around the head and mouth.

After feature extraction by the CLIP visual encoder and TED-Adapter, the class token of each frame was extracted as the frame-level global feature. The sequence of frame-level features for *T* consecutive frames is denoted as

(8) $$X=[x_{1},x_{2},\ldots ,x_{T}].$$

**Figure 4:**
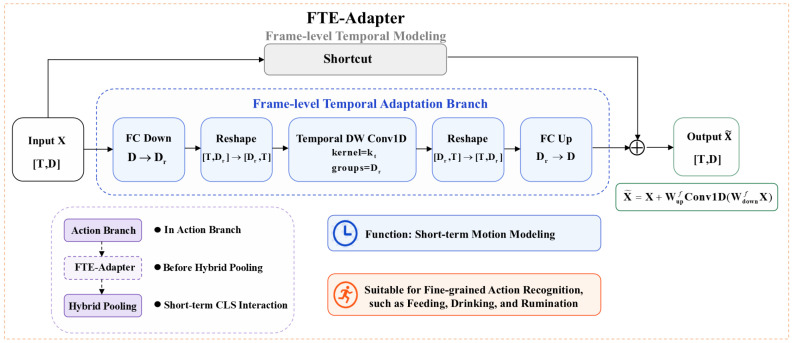
Structure of the FTE-Adapter.

As shown in Figure 4, FTE-Adapter was introduced into the action branch because feeding, drinking, and rumination depend strongly on short-term frame-level dynamics. It operates on the 512-dimensional projected class-token feature of each frame. The features were projected into a 256-dimensional bottleneck, reshaped from [B×T,512] to [B,256,T], processed by a depthwise one-dimensional convolution with kernel size 3, stride 1, padding 1, and 256 groups, and then restored to 512 dimensions:

(9) $$\tilde{X}=X+W_{\mathrm{up}}^{f}\mathrm{Conv}1D\left(W_{\mathrm{down}}^{f}X\right),$$

where $W_{\mathrm{down}}^{f}$ and $W_{\mathrm{up}}^{f}$ denote learnable down-projection and up-projection parameters, respectively. After frame-level enhancement, hybrid temporal pooling was used to aggregate the sequence into a video-level action representation. First, temporal average pooling was applied.

(10) $$x_{\mathrm{avg}}=\frac{1}{T}\sum_{t=1}^{T}{\tilde{x}}_{t}.$$

Subsequently, a temporal attention module was used to assign adaptive weights to different frames:

(11) $$\alpha_{t}=\frac{\exp(\mathrm{MLP}\left({\tilde{x}}_{t}\right))}{\sum_{j=1}^{T}\exp\left(\mathrm{MLP}\left({\tilde{x}}_{j}\right)\right)},\;x_{\mathrm{att}}=\sum_{t=1}^{T}\alpha_{t}{\tilde{x}}_{t}.$$

Finally, the average-pooled and attention-pooled features were fused using fixed weights:

(12) $$x_{a}=0.7x_{\mathrm{avg}}+0.3x_{\mathrm{att}}.$$

Here, ${\tilde{x}}_{t}$ denotes the enhanced action feature of the *t*-th frame, $\alpha_{t}$ denotes the attention weight assigned to that frame, and $x_{a}$ denotes the video-level action representation fed into the action classification head. The fixed fusion coefficients were set to 0.7 for average pooling and 0.3 for attention pooling in all experiments. Average pooling preserves the overall behavior state of the clip, whereas attention pooling emphasizes key frames containing discriminative action cues.

#### 3.2.2. Posture–Action Dual-Head Modeling and Behavioral Prior Loss

Dairy cow behavior can be interpreted at two semantic levels: posture state and action behavior. Standing and lying describe body posture states, whereas feeding, drinking, and rumination describe action behaviors that may co-occur with different posture states. Based on this characteristic, separate posture and action branches were constructed so that these two semantic levels could be modeled independently while their relationship was still preserved.

For the posture branch, the temporal average of frame-level class tokens was used as the video-level posture feature:

(13) $$x_{p}=\frac{1}{T}\sum_{t=1}^{T}x_{t}.$$

The posture prediction was then obtained as

(14) $$p^{p}=\mathrm{Softmax}\left(W_{p}x_{p}+b_{p}\right).$$

For the action branch, the video-level action feature generated by the temporal modeling module was used for classification:

(15) $$p^{a}=\mathrm{Softmax}\left(W_{a}x_{a}+b_{a}\right).$$

The posture and action losses were defined as cross-entropy losses:

(16) $$L_{p}=\mathrm{WCE}\left(p^{p},y^{p}\right),\;L_{a}=\mathrm{WCE}\left(p^{a},y^{a}\right),$$

where $y^{p}$ and $y^{a}$ denote the posture and action labels, respectively. In the experiments, WCE(·) denotes class-weighted cross-entropy, which was used to mitigate the influence of imbalanced class distributions. Through the dual-head prediction structure, spatial posture features and temporal action features can be learned separately. Consequently, interference between different semantic levels is reduced, and a structural basis is provided for the subsequent posture–action behavioral prior constraint.

To further constrain the plausible combinations between posture and action, a rule matrix R was constructed according to dairy cow behavioral priors:

(17) $$R=\left[\begin{array}{cccc} 0 & 0 & 0 & 0\\ 0 & 1 & 1 & 0 \end{array}\right].$$

The rows of the matrix correspond to standing and lying, whereas the columns correspond to none, feeding, drinking, and rumination. A value of 1 indicates an empirically low-probability posture–action combination; in this study, the constraint was mainly imposed on lying with feeding and lying with drinking. These relationships should be interpreted as probabilistic behavioral tendencies rather than absolute biological rules. Although lying with feeding or drinking is uncommon under conventional barn conditions, such combinations may occur because of illness, injury, stress, transitional movements, unusual facility layouts, or annotation uncertainty. Therefore, these combinations were neither removed from the dataset nor prohibited during inference. They were only assigned soft penalties during training, allowing sufficiently strong visual evidence to override the prior constraint. For the *n*-th sample, let the predicted posture probability be $p_{n}^{p}$ and the predicted action probability be $p_{n}^{a}$. The behavioral prior loss is defined as

(18) $$L_{r}=\frac{1}{N}\sum_{n=1}^{N}\sum_{i}\sum_{j}R_{ij}p_{n,i}^{p}p_{n,j}^{a}.$$

In this task, because the behavioral prior was mainly used to constrain lying with feeding and lying with drinking, the rule loss can be further written as

(19) $$L_{r}=\frac{1}{N}\sum_{n=1}^{N}p_{n,\mathrm{lying}}^{p}\left(p_{n,\mathrm{feeding}}^{a}+p_{n,\mathrm{drinking}}^{a}\right).$$

This loss can be regarded as a soft penalty on low-probability posture–action combinations. When high probabilities are simultaneously assigned to the lying posture and to feeding or drinking actions, the rule loss is increased. Conversely, when the model output is more consistent with dairy cow behavioral patterns, this loss is reduced. Unlike hard rule-based post-processing, the proposed prior constraint is incorporated into the training process in a differentiable form and does not directly force the inference results to be modified.

#### 3.2.3. Training Objective and Inference Mapping

During training, posture recognition, action recognition, and behavioral prior constraints were optimized simultaneously. The posture and action branches were supervised using cross-entropy losses, whereas the behavioral prior loss was used to constrain low-probability combinations between posture states and action behaviors. The total loss is defined as

(20) $$L=\lambda_{p}L_{p}+\lambda_{a}L_{a}+\lambda_{r}L_{r},$$

where $L_{p}$, $L_{a}$, and $L_{r}$ denote the posture classification loss, action classification loss, and behavioral prior loss, respectively, and $\lambda_{p}$, $\lambda_{a}$, and $\lambda_{r}$ are the corresponding loss weights. In the experiments, $\lambda_{p}$ and $\lambda_{a}$ were fixed, whereas $\lambda_{r}$ was varied to analyze the influence of the behavioral prior loss; for simplicity, $\lambda_{r}$ is denoted as λ in the Results section. Through this joint optimization objective, posture states, action dynamics, and posture–action compositional relationships can be learned simultaneously.

During inference, posture and action probability distributions were output by the model, and the corresponding predicted classes were obtained as follows:

(21) $${\hat{y}}^{p}=\arg\max_{i}p_{i}^{p},\;{\hat{y}}^{a}=\arg\max_{j}p_{j}^{a}.$$

Here, ${\hat{y}}^{p}$ denotes the predicted posture class, taking the value standing or lying, and ${\hat{y}}^{a}$ denotes the predicted action class, taking the value none, feeding, drinking, or rumination. The complete behavioral output of the model is represented not as a single five-class label but as a posture–action combination:

(22) $$\hat{y}=\left({\hat{y}}^{p},{\hat{y}}^{a}\right).$$

When the action prediction is none, the combination indicates that the target cow is only in a certain posture state. When the action prediction is feeding, drinking, or rumination, both the posture state and the action behavior are preserved in the output, such as standing with feeding or lying with rumination. For comparison with conventional five-class single-label classification models, when none is predicted by the action branch, the five-class label is determined by the posture branch and mapped to standing or lying; when feeding, drinking, or rumination is predicted by the action branch, the five-class label is mapped to the corresponding action category. This mapping is used only for performance comparison and does not alter the actual posture–action combination output of the proposed model.

### 3.3. Dataset and Data Reconstruction

The experiments were conducted using the publicly available CBVD-5 dairy cow behavior dataset [25]. According to the original publication, CBVD-5 was collected at a single ranch from 107 approximately 20-month-old dairy cows under an approximately 80% stocking density. More than 96 h of footage were recorded by seven fixed surveillance cameras under varying illumination conditions. The complete dataset contains 687 video segments and approximately 206,100 frames covering five behaviors, namely standing, lying, feeding, drinking, and rumination. The original annotations follow an AVA-style spatio-temporal action-detection format, in which each row records a video identifier, timestamp, bounding-box coordinates, behavior category, and target category.

**Figure 5:**
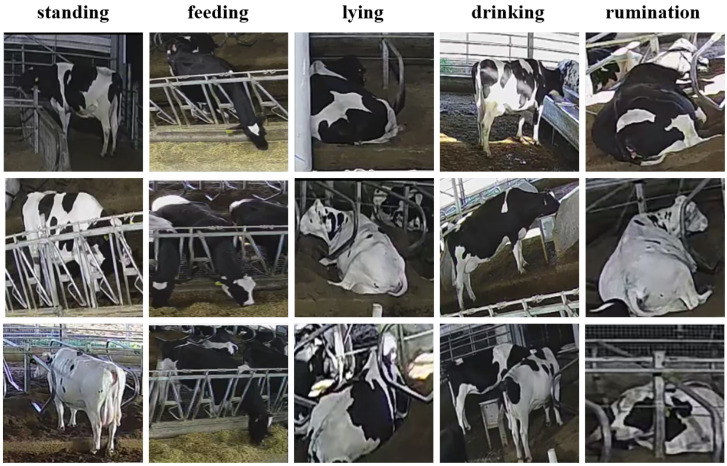
Representative examples of the five behavior categories in the reconstructed CBVD-5 dataset. Three examples are provided for each category, including standing, lying, feeding, drinking, and rumination, to illustrate the visual variability of dairy cow behaviors under real barn conditions.

Representative samples of the reconstructed clip-based classification dataset are shown in Figure 5. Three examples are provided for each final behavior category to illustrate intra-class variations under real barn conditions, including differences in body orientation, illumination, occlusion, and background complexity.

**Figure 6:**
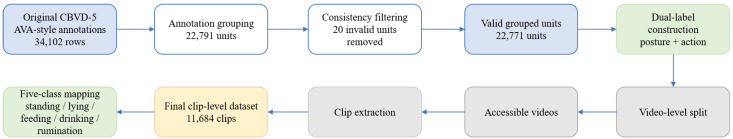
Workflow of dataset reconstruction from the original AVA-style CBVD-5 annotations to the reconstructed clip-level classification dataset.

The main reconstruction procedure from the original AVA-style annotations to the final clip-level classification dataset is summarized in Figure 6.

Because the original detection annotations could not be directly used for the clip-level dual-head classification task, a reconstruction procedure was performed. As shown in Figure 6, 34,102 annotation rows associated with 492 video identifiers were first grouped by video identifier, timestamp, bounding box, and target category, resulting in 22,791 annotation units. Each unit was then converted into a posture label, standing or lying, and an action label, none, feeding, drinking, or rumination. The none action label was assigned only when no explicit feeding, drinking, or rumination annotation was present. Twelve units with conflicting posture labels and eight units without a valid posture label were excluded, leaving 22,771 valid grouped units. Low-probability combinations were not filtered out during reconstruction; specifically, 33 lying–feeding units were retained and no lying–drinking unit was found. Thus, the behavioral prior was used only as a soft training constraint.

To avoid information leakage, the split was performed at the source-video level before clip generation. Using a fixed random seed of 42, the 492 annotated video identifiers were divided into 295 training, 98 validation, and 99 test identifiers by stratification according to the dominant posture–action combination in each video. All annotation units from the same source video were assigned to only one subset. Before clip extraction, the training, validation, and test subsets contained 13,409, 4456, and 4906 valid grouped units, respectively.

During clip extraction, only units with accessible source video segments in the downloaded dataset copy were retained. Finally, 11,684 clips were generated, including 7391 training clips, 2224 validation clips, and 2069 test clips from 160, 45, and 46 accessible source videos, respectively. For each retained unit, a 3-s clip was extracted around the annotated timestamp. The target cow was cropped according to the annotated bounding box, which was expanded by factors of 1.20 and 1.15 in the horizontal and vertical directions, padded to a square shape, and resized to 224×224 pixels. Eight frames were uniformly sampled from each generated clip during model loading. Therefore, the reported results correspond to behavior recognition under annotation-based target localization; object detection and multi-object tracking were not included in the current pipeline and remain necessary for fully end-to-end barn deployment.

For the proposed model, each training-list entry contained a clip path, a posture label, and an action label. For comparison with conventional five-class classifiers, the dual labels were deterministically mapped to a single five-class label, as summarized in Table 1.

During five-class evaluation, samples with the none action label were mapped to standing or lying according to the posture label, whereas samples labeled as feeding, drinking, or rumination were mapped directly to the corresponding action category. The same deterministic rule was applied to the dual-head predictions during evaluation.

**Figure 7:**
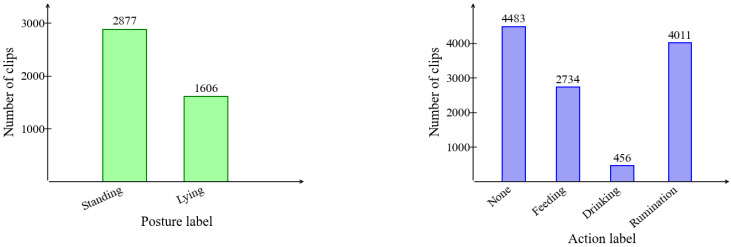
Posture-state and action-label distributions in the reconstructed CBVD-5 clip-based classification dataset. In the action distribution, the none class corresponds to posture-only samples without explicit feeding, drinking, or rumination annotations.

As summarized in Table 2, the reconstructed dataset is clearly imbalanced, with drinking being the least represented behavior in every subset. The overall posture-state and action-label distributions are further visualized in Figure 7. A class-weighting strategy was therefore adopted during training. Although the original CBVD-5 publication reports one ranch, 107 cows, and seven cameras for the complete dataset, the released annotations used here did not provide persistent individual identities or an explicit camera-to-video mapping. Therefore, animal-level and camera-level independence could not be further verified for each reconstructed subset.

### 3.4. Experimental Settings and Evaluation Metrics

CLIP ViT-B/16 was adopted as the visual encoder [27,35] and initialized using the official OpenAI CLIP ViT-B/16 pretrained weights. TED-Adapter, FTE-Adapter, and hybrid temporal pooling were introduced for video temporal modeling. During training, the main parameters of the CLIP visual encoder were frozen, and only the TED-Adapter, FTE-Adapter, temporal attention module, posture classification head, and action classification head were updated. For each video clip, eight frames (T=8) were uniformly sampled as the model input. AdamW was used as the optimizer [36]. The learning rate was 0.0003, the weight decay was set to 0.03, the batch size was set to 8, the total number of training epochs was set to 50, and the warmup epoch was set to 2. Data augmentation strategies, including RandAugment, were applied during training so that model generalization under complex barn environments could be improved. To maintain a consistent evaluation protocol, all comparison models and the proposed model were evaluated using the same training, validation, and test splits, the same number of sampled input frames (T=8), and the same major training settings, while differences in pretraining and model architecture were explicitly acknowledged in the Results section. To examine the stability of the proposed method, additional runs were conducted using three random seeds, namely 42, 3407, and 2026, while keeping the data split, input-frame setting, and training protocol unchanged. To mitigate class imbalance, weighted cross-entropy losses were applied separately to the posture and action branches. The posture-class weights for standing and lying were set to 0.90 and 1.10, respectively, whereas the action-class weights for none, feeding, drinking, and rumination were set to 0.60, 1.10, 3.00, and 1.00, respectively. Rather than being calculated directly using an inverse-frequency formula, these weights were selected empirically with reference to the training-set class distribution and were kept fixed across all dual-head experiments. In the total loss, $\lambda_{p}$ and $\lambda_{a}$ were fixed at 0.2 and 0.8, respectively, whereas the behavioral prior loss weight $\lambda_{r}$ was compared under different settings, including 0, 0.05, and 0.10; according to the experimental results, the behavioral prior loss weight was finally set to $\lambda_{r}=0.10$. All experiments were implemented using PyTorch 2.0.0 and conducted on an NVIDIA RTX A6000 GPU with CUDA 11.7.

To comprehensively evaluate model performance, Accuracy, Precision, Recall, and Macro-F1 were calculated for both the posture recognition task and the action recognition task. For the posture branch, the two categories were standing and lying, whereas for the action branch, the four categories were none, feeding, drinking, and rumination. Precision, Recall, and F1-score were first calculated for each category, and the corresponding macro-averaged metrics were then obtained by averaging the values over all categories with equal weight. Therefore, Macro-F1 was adopted as an important evaluation metric because it can better reflect the recognition performance of minority categories under imbalanced class distributions.

To facilitate comparison with conventional single-output five-class behavior recognition models, the dual-head outputs were further mapped to five-class behavior predictions during evaluation. Specifically, when the action branch predicted none, the final five-class label was determined by the posture branch and was assigned as either standing or lying. When the action branch predicted feeding, drinking, or rumination, the corresponding action category was directly used as the final five-class prediction, regardless of the posture prediction. Based on this mapping rule, each test sample was assigned one of five final behavior labels, namely standing, lying, feeding, drinking, and rumination. Five-class Accuracy, Macro Precision, Macro Recall, and Macro-F1 were then calculated from the mapped five-class predictions and the corresponding five-class ground-truth labels.

## 4. Results

### 4.1. Comparison with Existing Video Recognition Models

The five-class behavior recognition performance of different methods on the test set is reported in Table 3. R(2+1)D-R34 [20], SlowFast-R50 [21], and Video Swin-T [24] were selected as representative traditional video behavior recognition models. These models cover three typical video-recognition architectures, namely spatiotemporal convolutional networks, two-pathway temporal modeling networks, and Transformer-based video models. In this study, the three comparison models were trained from scratch without pretrained weights, and their backbones were not frozen; therefore, their total parameters were also their trainable parameters. These comparison methods were implemented under a single-output five-class classification formulation, whereas the proposed model was designed with a posture–action dual-head output structure. To ensure that a consistent evaluation protocol was maintained, the dual-head predictions were mapped to five behavior categories during testing. Specifically, when no obvious action was predicted by the action branch, the final category was determined by the posture branch and was assigned as either standing or lying. When feeding, drinking, or rumination was predicted by the action branch, the corresponding action category was used as the final five-class result. This mapping was conducted only for comparison with single-output models under five-class evaluation metrics, while the posture and action predictions were still retained by the proposed model.

As shown in Table 3, the proposed method achieved a five-class behavior recognition accuracy of 75.45% and a Macro-F1 of 0.7246 on the test set. Compared with R(2+1)D-R34, the accuracy was increased by 4.30 percentage points, and the Macro-F1 was improved by 0.0535. Compared with Video Swin-T, the accuracy and Macro-F1 were improved by 4.11 percentage points and 0.0514, respectively. Compared with SlowFast-R50, the accuracy and Macro-F1 were improved by 1.69 percentage points and 0.0096, respectively. In the additional three-seed runs, the final five-class recognition performance varied by approximately 0.6 percentage points, indicating that the proposed method was relatively stable under the implemented experimental protocol. These results suggest that, on the reconstructed CBVD-5 clip-level test set, fine-grained dairy cow behaviors can be more effectively recognized when CLIP visual representations are combined with temporal adaptation and pooling mechanisms designed for video sequences. From the perspective of model parameters, the proposed method contains 115.088 M parameters in total because it uses the CLIP visual encoder as the backbone. However, the CLIP visual backbone was frozen during training, and only the TED-Adapter, FTE-Adapter, temporal attention module, and classification heads were updated. Consequently, the number of trainable parameters was 28.895 M. In contrast, the three traditional video comparison models were trained from scratch without frozen backbones, so all their parameters were trainable. Therefore, the parameter comparison should be interpreted primarily from the perspective of trainable parameters and training cost rather than total model size. Under this setting, the proposed method required fewer trainable parameters than R(2+1)D-R34 (63.557 M) and SlowFast-R50 (33.655 M), and a number close to Video Swin-T (27.854 M), while maintaining competitive five-class behavior recognition performance. It should be noted that the proposed method was initialized using a large-scale pretrained CLIP visual encoder, whereas R(2+1)D-R34, Video Swin-T, and SlowFast-R50 were trained from scratch in the present experiments. Therefore, the comparison reflects performance under the implemented training protocol rather than an architecture-level claim of absolute superiority. The results mainly indicate that the proposed adaptation strategy is effective when pretrained CLIP representations are available for the reconstructed CBVD-5 task. Future work will further include pretrained versions of representative video recognition models to provide a more strictly controlled comparison.

### 4.2. Ablation Experiments on Main Components

To verify the effectiveness of the proposed temporal modeling modules, a baseline model was first constructed using the CLIP visual encoder, temporal average pooling, and a dual-branch classifier. On the basis of this baseline, the token-level temporal adaptation module and the frame-level temporal module, consisting of FTE-Adapter and hybrid temporal pooling, were separately introduced for ablation comparison. The dual-head design was not adopted merely to improve absolute five-class accuracy, but to explicitly separate posture states from action behaviors. This decomposition is consistent with the semantic structure of dairy cow behaviors and provides the basis for the behavioral prior loss, because low-probability posture–action combinations can only be softly constrained when posture and action probabilities are predicted separately. The experimental results are presented in Table 4.

As shown in Table 4, the CLIP + Mean Pooling baseline obtained a Posture Macro-F1 of 0.9352. However, its Action Macro-F1 was only 0.4338, and the mapped five-class Macro-F1 was only 0.3733. This result indicates that simple averaging of CLIP frame-level features can preserve certain static posture information, but continuous dynamic variations involved in action categories such as feeding, drinking, and rumination cannot be sufficiently represented. When only the frame-level temporal module was introduced, the Action Macro-F1 increased to 0.6197, and the five-class Macro-F1 increased to 0.5511. This improvement suggests that short-term inter-frame relationships and key action frames can be more effectively modeled when FTE-Adapter and hybrid temporal pooling are combined, thereby enhancing the discriminative capacity of the action branch.

When only the token-level temporal adaptation module was incorporated, the Posture Macro-F1 and Action Macro-F1 increased to 0.9835 and 0.7263, respectively, while the five-class accuracy and five-class Macro-F1 reached 72.45% and 0.6613, respectively. The magnitude of improvement was greater than that obtained by introducing only the frame-level temporal module. This indicates that dynamic relationships between adjacent frames can be more comprehensively modeled when token-level temporal interaction is introduced during CLIP feature extraction. When token-level temporal adaptation and the frame-level temporal module were introduced simultaneously, the Posture Macro-F1, Action Macro-F1, five-class accuracy, and five-class Macro-F1 reached 0.9850, 0.7455, 73.85%, and 0.7019, respectively, outperforming the configurations in which either module was used alone. These results demonstrate that the two temporal modeling strategies are complementary: token-level temporal adaptation emphasizes local temporal interaction during visual encoding, whereas the frame-level temporal module strengthens short-term action dynamics and highlights key action frames at the video-level representation stage. Their combination therefore enables further improvement in overall recognition performance.

### 4.3. Analysis of the Behavioral Prior Loss Weight

With the temporal modeling modules kept unchanged, different values of the behavioral prior loss weight λ were compared to further examine its influence on model performance, as shown in Table 5. This experiment was designed to evaluate how posture–action prior constraints affected the action branch and the final five-class behavior recognition results.

When λ=0.00, the behavioral prior loss was disabled, and the model was therefore equivalent to the ablation configuration in which both TED-Adapter and the frame-level module were included without posture–action prior constraints.

As shown in Table 5, when λ=0.00, namely when the behavioral prior loss was not applied, an Action Macro-F1 of 0.7455 was achieved, while the five-class accuracy and five-class Macro-F1 were 73.85% and 0.7019, respectively. The effect of the behavioral prior loss was modest and should be interpreted cautiously. When λ=0.05, the Action Macro-F1 decreased from 0.7455 to 0.7383, whereas the mapped five-class Macro-F1 increased from 0.7019 to 0.7202. This trade-off occurs because the final five-class prediction is obtained from the joint posture–action outputs rather than from the action branch alone. The action metric evaluates four action classes independently, including the none class, whereas the five-class metric further depends on how the posture and action outputs are combined. Therefore, a weak prior constraint may slightly change the action-branch decision distribution while reducing selected inconsistent posture–action combinations, leading to a better mapped five-class result. In this sense, the behavioral prior loss should be understood as a soft joint-consistency constraint rather than as a mechanism that necessarily improves independent action recognition. When λ=0.10, the Action Macro-F1 increased to 0.7605, and the five-class accuracy and five-class Macro-F1 reached 75.45% and 0.7246, respectively, corresponding to the best overall performance among the reported settings. Therefore, λ=0.10 was finally adopted as the behavioral prior loss weight.

### 4.4. Per-Class Five-Class Performance

Because the reconstructed dataset was imbalanced, the per-class performance of the final mapped five-class predictions was analyzed for the model with λ=0.10. For reference, within the action branch, the none class achieved a Precision of 0.8282, a Recall of 0.6412, and an F1-score of 0.7228.

Table 6 reports the per-class results after the dual-head outputs were mapped to the final five behavior categories. Standing and feeding achieved F1-scores of 0.8394 and 0.8285, respectively. In contrast, lying produced the lowest Recall and F1-score, at 0.3705 and 0.4642. Of the 332 lying samples, 205 were mapped incorrectly to rumination. This confusion is attributable to the compositional definition of the final labels: both lying-only and lying–rumination samples exhibit a lying posture, while their distinction depends primarily on weak mouth and jaw movements. Such subtle cues can be obscured by occlusion, limited image resolution, and complex backgrounds, causing the action branch to predict rumination for some lying-only clips. Drinking was represented by only 62 test samples but achieved an F1-score of 0.7302. Although this result indicates useful recognition capability for the minority class, its statistical stability and generalizability remain limited by the small support and should be interpreted cautiously.

**Figure 8:**
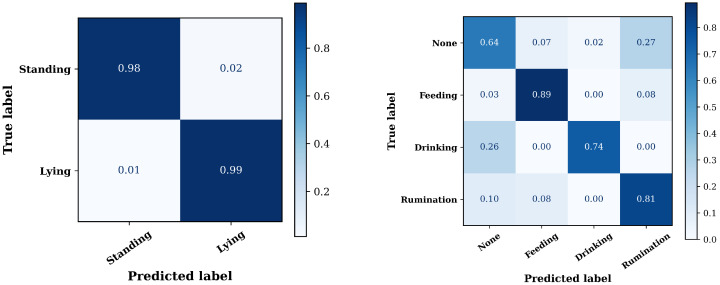
Normalized confusion matrices of the posture and action branches on the test set. The left matrix shows the posture branch for standing and lying, and the right matrix shows the action branch for none, feeding, drinking, and rumination.

To further visualize the class-wise recognition behavior of the proposed model, normalized confusion matrices of the posture and action branches are presented in Figure 8. The posture branch shows a clear diagonal distribution, with normalized correct classification rates of 0.98 for standing and 0.99 for lying, indicating that global posture states can be recognized reliably. For the action branch, feeding, drinking, and rumination achieve normalized correct classification rates of 0.89, 0.74, and 0.81, respectively. The main confusions occur between none and rumination and between drinking and none, which suggests that weak mouth movements, short drinking events, and partial occlusion may still make fine-grained action discrimination difficult under real barn conditions.

## 5. Discussion

The improvement achieved by the proposed method mainly results from the combination of pretrained visual representation, temporal adaptation, and behavioral prior constraints. The CLIP visual encoder provides strong frame-level appearance features for describing body contour, posture state, and the spatial relationship between the cow and surrounding facilities such as the feed trough and water trough. However, fine-grained behaviors such as feeding, drinking, and rumination often share similar head-lowering postures and mouth-related movements, making them difficult to distinguish using static appearance alone. Therefore, TED-Adapter, FTE-Adapter, and hybrid temporal pooling were introduced to enhance dynamic information across consecutive frames and capture short-term local motion changes more effectively.

Specifically, TED-Adapter is inserted into the CLIP visual Transformer blocks and mainly models temporal changes and adjacent-frame difference information at the token level, which is helpful for capturing subtle movements in small regions such as the head, mouth, and neck. FTE-Adapter operates on the frame-level class-token sequence in the action branch and works with average pooling and attention pooling to obtain a video-level action representation that preserves the overall behavioral state while emphasizing key action frames. The behavioral prior does not guarantee biologically plausible predictions; it only reduces two selected low-probability combinations, namely lying with feeding and lying with drinking. Behavior duration, state transitions, individual differences, and broader ethological rules are not modeled. Its effect should therefore be interpreted as a limited soft consistency constraint rather than comprehensive behavioral reasoning.

In terms of category-level performance, the separate posture branch recognized standing and lying reliably because these states are mainly determined by global body configuration. However, the mapped five-class lying category remained difficult because lying-only clips had to be distinguished from lying–rumination clips through the action prediction. The low lying Recall of 0.3705 therefore reflects uncertainty in detecting subtle rumination movements rather than a failure to recognize the lying posture itself. Drinking was also challenging because it had only 62 samples in the test set and may resemble feeding when the cow’s head is close to a trough. Rumination may likewise be confused with posture-only or other head-related behaviors when mouth movement is weak or partially occluded.

Several limitations remain. First, the dataset is clearly imbalanced, and the shortage of minority-class samples limits the model’s ability to learn drinking-related patterns. Second, severe occlusion, overlapping cows, bounding-box errors, complex backgrounds, and illumination variation may still affect recognition stability. A further limitation concerns the dependence on pre-localized target-cow regions. Because bounding-box annotations were used to generate the cropped clips in the present experiments, recognition errors caused by automatic detection, identity switching, missed targets, and inaccurate tracking were not evaluated. In a practical barn, occlusion, overlapping animals, illumination changes, and changing camera viewpoints may propagate localization errors to the behavior recognition stage. Third, the experiments were mainly conducted on a reconstructed clip-level dataset, and generalization across farms, camera views, seasons, breeds, and management conditions still requires further validation. In addition, the behavioral prior used here encodes only two low-probability posture–action combinations and should not be regarded as a complete ethological model. Although its soft formulation reduces the risk of enforcing an incorrect rule, it may still bias predictions when cows exhibit abnormal or context-dependent behaviors. Future work should learn context-aware priors from larger multi-farm datasets and incorporate behavior duration and state-transition information. It should also integrate cow detection, multi-object tracking, behavior recognition, multimodal perception, and real-time inference into an end-to-end pipeline. The complete system should be evaluated on continuous, untrimmed barn videos in terms of recognition accuracy, computational efficiency, communication efficiency, and privacy-aware deployment [37,38].

## 6. Conclusions

In this study, a dairy cow behavior recognition method integrating temporal modeling and behavioral priors was proposed. The method used the CLIP visual encoder, temporal average pooling, and a dual-branch classifier as the baseline, and further introduced TED-Adapter, FTE-Adapter, hybrid temporal pooling, and behavioral prior loss to enhance temporal dynamic modeling across consecutive video frames and constrain low-probability posture–action combinations. To evaluate the effectiveness of the proposed method, three groups of experiments were conducted. First, comparison experiments with representative video recognition models, including R(2+1)D-R34, Video Swin-T, and SlowFast-R50, were performed to assess overall five-class behavior recognition performance. Second, ablation experiments were designed to analyze the contributions of TED-Adapter, FTE-Adapter, and the frame-level temporal modeling module. Third, experiments with different behavioral prior loss weights were conducted to examine the effect of posture–action prior constraints on recognition performance. The proposed method achieved a five-class accuracy of 75.45%, a five-class Macro-F1 of 0.7246, and an Action Macro-F1 of 0.7605 on the test set. Compared with the CLIP + Mean Pooling baseline, the five-class accuracy and Macro-F1 were improved by 16.68 percentage points and 0.3513, respectively. Compared with R(2+1)D-R34, Video Swin-T, and SlowFast-R50, the proposed method improved five-class accuracy by 4.30, 4.11, and 1.69 percentage points, respectively, and improved five-class Macro-F1 by 0.0535, 0.0514, and 0.0096, respectively. These results demonstrate that the proposed method improved fine-grained dairy cow behavior recognition on the reconstructed CBVD-5 clip-level test set. However, because the current evaluation was based on data collected from a single ranch and annotation-based target localization, further validation across independent farms, cow populations, camera systems, seasons, and end-to-end detection and tracking pipelines is required before practical deployment.

## Figures and Tables

**Table 1:** Posture–action label mapping for five-class evaluation.

Behavior Annotation	Posture Branch	Action Branch	Five-Class Label
Standing only	0: standing	0: none	standing
Lying only	1: lying	0: none	lying
Standing + feeding	0: standing	1: feeding	feeding
Standing + drinking	0: standing	2: drinking	drinking
Standing + rumination	0: standing	3: rumination	rumination
Lying + rumination	1: lying	3: rumination	rumination

**Table 2:** Five-class behavior distribution of the reconstructed clip-based dataset.

Subset	Standing	Lying	Feeding	Drinking	Rumination	Total
Training set	1847	1030	1784	286	2444	7391
Validation set	565	244	501	108	806	2224
Test set	465	332	449	62	761	2069
Total	2877	1606	2734	456	4011	11,684

**Table 3:** Comparison of performance and parameter numbers of different methods on the test set.

Method	5-Class Acc	Macro Precision	Macro Recall	Macro-F1	Total	Trainable
R(2+1)D-R34	71.15%	0.6681	0.6803	0.6711	63.557 M	63.557 M
Video Swin-T	71.34%	0.6942	0.6651	0.6732	27.854 M	27.854 M
SlowFast-R50	73.76%	0.7043	0.7470	0.7150	33.655 M	33.655 M
Proposed method	75.45%	0.7423	0.7236	0.7246	115.088 M	28.895 M

**Table 4:** Effects of different model components on recognition performance.

Baseline	TED-Adapter	Frame-Level Module	Posture Macro-F1	Action Macro-F1	5-Class Acc	5-Class Macro-F1
✓	×	×	0.9352	0.4338	58.77%	0.3733
✓	×	✓	0.9414	0.6197	62.45%	0.5511
✓	✓	×	0.9835	0.7263	72.45%	0.6613
✓	✓	✓	0.9850	0.7455	73.85%	0.7019

**Table 5:** Effects of different behavioral prior loss weights λ on model performance.

λ	Posture Macro-F1	Action Macro-F1	5-Class Acc	5-Class Macro-F1
0.00	0.9850	0.7455	73.85%	0.7019
0.05	0.9869	0.7383	74.87%	0.7202
0.10	0.9830	0.7605	75.45%	0.7246

**Table 6:** Per-class five-class recognition performance of the final model on the test set.

Class	Precision	Recall	F1-Score	Support
Standing	0.8854	0.7978	0.8394	465
Lying	0.6212	0.3705	0.4642	332
Feeding	0.7726	0.8931	0.8285	449
Drinking	0.7188	0.7419	0.7302	62
Rumination	0.7135	0.8147	0.7607	761
Macro average	0.7423	0.7236	0.7246	–

## Data Availability

The CBVD-5 dataset used in this study is publicly available from the original publication. The processed lists and code are available from the corresponding author upon reasonable request.
